# Editorial: Assessment of nutritional status in chronic diseases

**DOI:** 10.3389/fnut.2025.1662137

**Published:** 2025-08-13

**Authors:** Olivia Di Vincenzo, Mariacristina Siotto

**Affiliations:** ^1^Department of Experimental Medicine, Sapienza University of Rome, Rome, Italy; ^2^IRCCS Fondazione Don Carlo Gnocchi ONLUS, Florence, Italy

**Keywords:** nutrition, clinical nutrition, malnutrition, nutritional risk, inflammation, illnesses, dietary intake, patients

The role of nutrition in chronic disease management has gained substantial attention, given its impact on clinical outcomes, disease progression, and patients' quality of life, including mental health. Across a wide range of conditions, from kidney and respiratory diseases to neurodegenerative and oncological disorders, emerging evidence underscores the intricate interplay between dietary intake, nutritional status, and overall systemic health.

This Research Topic includes recent studies examining crucial aspects of nutrition in chronic illness. It highlights novel factors related to nutrition, advanced assessment tools, and prognostic indicators that improve the understanding of how nutrition affects the course of health and disease. It includes 21 original research articles, one systematic review, and one case report, offering diverse and clinically relevant perspectives on the integration of nutritional care into chronic disease management.

Dietary intake and adherence are fundamental components of nutritional care, particularly in populations with chronic conditions. Several studies provide valuable insights into the associations between specific dietary patterns and clinical outcomes. In patients undergoing hemodialysis, higher consumption of animal protein was linked to improved sleep quality and better overall quality of life, as reported by Jafari Maskouni et al.. In individuals with chronic obstructive pulmonary disease (COPD), Fu et al. analyzed data from NHANES 1999–2018 and found that higher dietary intake of niacin was significantly associated with reduced all-cause and cardiovascular mortality over an average follow-up of 8.3 years, suggesting a potentially protective role of this nutrient in COPD prognosis and management. Nutritional intake also appears to play a role in mental health among individuals with chronic kidney disease (CKD). The study by Yu et al., based on NHANES 2007–2014 data, demonstrated that higher intakes of vitamins A, B1, B6, D, and K were each linked to a significantly lower risk of depression. Moreover, when evaluating the joint effect of nine essential vitamins (A, B1, B2, B6, B12, C, D, E, and K), a synergistic protective association emerged, with vitamin A contributing most prominently. Lastly, in individuals with type 2 diabetes, Atinafu and Tilahun reported relatively high adherence to dietary recommendations, reinforcing the value of personalized dietary education and sustained counseling efforts to promote long-term adherence and improve clinical outcomes in this population.

The role of mineral homeostasis and nutritional status have been evaluated in various chronic conditions. In a comprehensive national survey analysis by Xia et al., the Magnesium Depletion Score (MDS), an index reflecting systemic magnesium deficiency, was strongly associated with both the prevalence and mortality of urinary incontinence (UI) subtypes, including stress, urgency, and mixed forms. Higher MDS not only correlated with increased severity of UI but also independently predicted all-cause mortality, underscoring magnesium's potential influence on urinary tract health and overall prognosis. Similarly, an analysis by Cao H et al. using NHANES data revealed a protective relationship between higher serum calcium levels and the risk of periodontal disease. Individuals with higher serum calcium exhibited a significantly reduced likelihood of periodontitis. Complementing these mineral-focused findings, Cao X et al. investigated the association between magnesium depletion and gout risk in a large adult population. The study demonstrated a clear positive relationship between higher magnesium depletion and increased gout prevalence, independent of dietary magnesium intake. This highlights the potential role of systemic magnesium deficiency, rather than insufficient intake, in the pathogenesis of gout, suggesting the importance of assessing magnesium status in the management of hyperuricemia-related disorders.

Nutritional status has been assessed through anthropometric, body composition, and functional measures across diverse clinical populations, revealing critical insights into health outcomes. Undernutrition is a major concern among adolescents; for instance, as shown by Zafar et al., the high prevalence of underweight among school-going girls in Pakistan, highlights the urgent need for targeted nutritional education and policy interventions to promote healthy dietary habits and ensure equitable access to safe and nutritious food. In pediatric populations, alterations in body composition can indicate early cardiometabolic risk: longitudinal data by Jin et al. indicated that increasing waist circumference and waist-to-height ratio trajectories in children were associated with higher left ventricular mass index and a greater likelihood of developing left ventricular hypertrophy, underscoring the importance of early lifestyle interventions and continuous monitoring. Among older adults, functional measures such as handgrip strength (HGS) have been explored as potential indicators of nutritional status. In a study by Alamri and Simbawa in hospitalized geriatric patients, HGS was found to be influenced by factors such as advanced age, low hemoglobin, and elevated HbA1c. Although it is a sensitive tool in identifying malnutrition or risk of malnutrition, its low specificity limits its standalone use for diagnosis. Similarly, as reported by Kaluzniak-Szymanowska et al. in older patients with COPD, distinct body composition phenotypes, including sarcopenia, obesity, and sarcopenic obesity, were associated with greater disease severity and significantly impaired physical performance. These findings reinforce the need for comprehensive nutritional evaluations that go beyond traditional measures like body mass index (BMI). Furthermore, a study by Fernández-Jiménez et al. showed that in patients with idiopathic pulmonary fibrosis, sarcopenia was highly prevalent and associated to both disease severity and reduced quality of life. Notably, a combination of anthropometric and functional measures was effective in predicting sarcopenia and 1-year mortality risk, highlighting the importance of early, integrated assessments to inform timely and targeted interventions.

A systematic review by Mentxakatorre et al. highlighted the complex and clinically significant relationship between nutritional status and quality of life in patients with Parkinson's disease (PD). Both motor and non-motor symptoms were found to be influenced by nutritional status, with undernutrition or unintentional weight loss negatively affecting disease progression and functional independence. Additionally, PD treatments were shown to impact body weight, emphasizing the need for continuous nutritional monitoring. Notably, the review highlighted the emerging role of the gut-brain axis: adequate nutritional status was associated with a more balanced intestinal microbiota, which, in turn, was associated with slower cognitive decline, better performance in activities of daily living, and enhanced overall quality of life.

Nutritional status and its prognostic implications have also been assessed adopting several tools and/or indices across diverse clinical populations. These tools are being refined or complemented by emerging methodologies and integrated assessments. For example, the Global Leadership Initiative on Malnutrition (GLIM) criteria remain a cornerstone for malnutrition diagnosis, incorporating parameters such as weight loss, reduced food intake, and inflammation. Recent advances, such as the application of machine learning (Rischmüller et al.) in patients with chronic gastrointestinal diseases, have reaffirmed the value of GLIM while also highlighting additional relevant indicators, such as phase angle, skeletal muscle mass index, limb circumferences, and nutritional biomarkers like albumin and prealbumin, that enhance diagnostic accuracy. Comparative studies further emphasize the variability in malnutrition detection depending on the tools employed. In hospitalized older adults (da Silva et al.), the GLIM criteria identified significantly more malnourished individuals than the Mini Nutritional Assessment (MNA), with a notable proportion classified as severely malnourished. Both tools correlated with frailty and sarcopenia, though the MNA-functional form showed stronger associations, suggesting different clinical utilities in geriatric populations. Beyond diagnostic scope, several indices have demonstrated prognostic value in predicting adverse outcomes. The Prognostic Nutritional Index (PNI), which reflects immunonutritional status, was shown to be independently associated with lower all-cause and cardiovascular mortality among individuals with cardiovascular disease and (pre)diabetes in a large NHANES study by Xu et al.. Similarly, in gynecologic cancer survivors with lower limb lymphedema, the study by Zhu et al. showed that low PNI and serum albumin levels were prevalent and associated with poor nutritional status and anemia, underscoring the importance of routine nutritional monitoring in oncology follow-up care. In patients with CKD (Wang et al.), malnutrition often coexists with systemic inflammation and other metabolic disturbances. Predictive models have identified hypoalbuminemia risk factors, such as anemia, hyponatremia, and hypocalcemia, that can be used to guide early intervention strategies. Moreover, combined markers of nutritional and inflammatory status, such as the Advanced Lung Cancer Inflammation Index (ALI), have emerged as useful predictors of mortality in several chronic diseases. Among asthma and CKD populations (Li et al.), higher ALI scores were consistently associated with reduced risk of all-cause and cardiovascular mortality. In CKD (Zhou et al.), the prognostic significance was particularly notable when depression was also considered: individuals with poor nutritional-inflammatory status and coexisting depressive symptoms had markedly elevated mortality risks, emphasizing the need for integrated psychosocial and nutritional care. Other composite scores, such as the Naples Prognostic Score (NPS), have shown similar utility. As reported by Kang et al., in individuals with COPD, elevated NPS values were linked to greater disease susceptibility, impaired lung function, and increased mortality, especially in smokers. These findings highlight the NPS as a promising tool for early identification and risk stratification. The Oxidative Balance Score (OBS), which integrates dietary and lifestyle-related antioxidant/pro-oxidant exposures, was inversely associated with muscular dystrophy risk, as reported in the study by Tang et al.. This suggests a potential protective role of favorable oxidative balance, expanding the relevance of nutrition-related indices into the neuromuscular disease domain.

Finally, a case-report by Oliveira et al. describes the case of a 78-year-old underweight and weakened male with a history of ischemic stroke and multiple comorbidities. Over a 10-month follow-up period, a percutaneous endoscopic gastrostomy tube was placed, and nutritional management was tailored based on regular biochemical and nutritional assessments. Personalized nutritional intervention, including a caloric surplus and dietary modifications, led to weight gain, improved muscle mass, and enhanced biochemical blood parameters, highlighting the importance of comprehensive nutritional management in post-stroke patients to improve clinical outcomes and quality of life.

In summary, the articles included in this Research Topic highlight the increasingly recognized role of nutrition in the management of several chronic diseases, including renal, respiratory, neurodegenerative, and metabolic disorders. Together, these findings demonstrate how dietary intake, nutritional status, and tailored assessment tools not only inform clinical decision-making but also contribute to preventing complications, enhancing quality of life, and improving patient outcomes. Moving forward, the integration of evidence-based nutritional strategies into routine chronic care, supported by comprehensive, multidimensional assessments, will be essential in advancing more effective, personalized, and patient-centered healthcare models.

